# HiCBin: binning metagenomic contigs and recovering metagenome-assembled genomes using Hi-C contact maps

**DOI:** 10.1186/s13059-022-02626-w

**Published:** 2022-02-28

**Authors:** Yuxuan Du, Fengzhu Sun

**Affiliations:** Department of Quantitative and Computational Biology, University of Southern California, Los Angeles, USA

**Keywords:** Metagenomic Hi-C, Zero-inflated negative binomial normalization, Spurious contact detection, Leiden clustering, Potts model, Contig binning

## Abstract

Recovering high-quality metagenome-assembled genomes (MAGs) from complex microbial ecosystems remains challenging. Recently, high-throughput chromosome conformation capture (Hi-C) has been applied to simultaneously study multiple genomes in natural microbial communities. We develop HiCBin, a novel open-source pipeline, to resolve high-quality MAGs utilizing Hi-C contact maps. HiCBin employs the HiCzin normalization method and the Leiden clustering algorithm and includes the spurious contact detection into binning pipelines for the first time. HiCBin is validated on one synthetic and two real metagenomic samples and is shown to outperform the existing Hi-C-based binning methods. HiCBin is available at https://github.com/dyxstat/HiCBin.

**Supplementary Information:**

The online version contains supplementary material available at (10.1186/s13059-022-02626-w).

## Background

Microbial communities consist of a wide range of microorganisms with many unexploited enzymes and metabolic potentials encoded in the genomes of these diverse species [1, 2]. Traditional pure cultures grown in the laboratory are insufficient to explore the microbial diversity because most microorganisms cannot be cultivated [3, 4]. As a culture-independent genomic approach, metagenomics avoids the isolation or cultivation of microorganisms and provides a broad aspect of the community structure and the functional capabilities present in complex ecosystems [5, 6].

The employment of next-generation sequencing techniques revolutionized metagenomic studies. Genomic fragments are directly sequenced from the microbial ecosystems, generating huge amounts of short reads from various environments, such as the human gut, soil, and ocean water [7]. However, the information on the genome identity of reads is lost due to the randomness of the whole-genome shotgun sequencing (WGS) [8]. To retrieve such information, metagenomic analysis assembles the WGS reads into relatively longer contigs. Then, assembled contigs are clustered into metagenome-assembled genomes (MAGs) [9]. This process is usually referred to as metagenomic binning. Traditional shotgun-based binning approaches to the accurate retrieval of MAGs depend on the contig similarity measurements from GC-content, tetra-mer composition, and/or co-abundance feature of the contigs across multiple samples [10–14]. Although experiments have demonstrated that co-abundance profiles across a series of samples enable the discovery of new microbial organisms effectively [15], the requirement for enough samples to obtain reliable co-abundance relationships between contigs may not be satisfied due to the cost restriction and limited capability to collect samples, impairing the effectiveness of these conventional binning approaches.

High-throughput chromosome conformation capture (Hi-C) [16] is a DNA proximity ligation technique having the capacity to retrieve MAGs accurately from a single sample. Hi-C technique generates millions of paired-end reads linking DNA fragments in close proximity within cells and has already been utilized to explore topologically associated domains and the compartment property of the mammalian genomes [16, 17]. When applied to metagenomics (metagenomic Hi-C), Hi-C technique is combined with traditional shotgun sequencing and has shown great capabilities to the genome binning and the simultaneous retrieval of high-quality MAGs from a single sample [18, 19].

Metagenomic Hi-C-based binning analysis usually adheres to a standard procedure. Short reads are generated by shotgun sequencing from the microbial community sample. In parallel, paired-end Illumina Hi-C sequence reads are obtained from the same sample. Contigs are assembled from the shotgun sequencing reads, and paired-end Hi-C reads are mapped to the assembled contigs to generate raw contact maps, which are then normalized to correct the strong experimental biases. Finally, normalized contact maps are clustered to construct draft genomic bins. From this standard procedure, we can conclude that there are two vital steps directly influencing the binning performance: normalization and clustering. Indeed, several Hi-C-based binning pipelines have been developed using different strategies to do normalization and clustering. ProxiMeta [20], a commercial metagenomic genome binning platform, took the abundance of the contigs into account to normalize the raw Hi-C contacts and then clustered contigs into genome bins using a proprietary MCMC-based algorithm based on their Hi-C linkages [20]. MetaTOR [21] and bin3C [22] are two state-of-the-art open-source pipelines. MetaTOR divided the raw Hi-C contacts by the product of coverage of contig pairs and then applied the Louvain algorithm with the classical Newman-Girvan criterion to cluster contigs [21]. Besides the Hi-C data, MetaTOR can also process the meta3C datasets [23]. bin3C designed a two-stage normalization method to process the raw contact maps [22]. It first divided the raw Hi-C counts by the product of the number of restriction sites and then used the Knight-Ruiz algorithm [24] to construct a general doubly stochastic matrix. For the clustering step, bin3C utilized the Infomap software (v0.19.25) [25] to bin contigs. But comprehensive evaluations of different normalization methods and clustering algorithms for the metagenomic Hi-C binning remain sparse [26].

We recently put forward three explicit experimental biases for raw metagenomic Hi-C contacts: the number of restriction sites, contig length, and contig coverage, and demonstrated that normalization methods in publicly available Hi-C-based binning pipelines cannot correct all explicit biases [27]. Those pipelines implementing normalization by the number of restriction sites cannot even be carried out when the restriction enzymes employed in Hi-C experiments are not specified. Moreover, spurious inter-species contacts (Hi-C contacts between contig pairs from different species) derived from the ligation of DNA fragments between closely related species weakened the interpretability of the Hi-C data [28]. However, none of available Hi-C-based binning pipelines attempted to detect and remove the spurious contacts. As for the clustering step, though several community detection algorithms have been employed to cluster the contigs, those clustering algorithms were not sufficiently effective in general applications [26].

To solve these problems, we develop HiCBin, a new open-source metagenomic Hi-C-based binning pipeline, to recover high-quality MAGs. We employ HiCzin [27], a novel normalization method designed for metagenomic Hi-C contact maps, to remove the experimental biases. HiCBin also detects and removes the spurious inter-species contacts for the first time among all Hi-C-based binning pipelines. In the clustering step, the Leiden algorithm [29], an advanced modularity-based community detection algorithm, is introduced to the metagenomic binning domain. The Leiden algorithm has proved to be strongly preferable to one of the most popular community detection algorithms, the Louvain algorithm in the experimental benchmarks [29, 30]. We also select a general and flexible modularity function based on the Reichardt and Bornholdt’s Potts model [31, 32] to overcome the resolution limit of the classical Newman-Girvan criterion utilized in the MetaTOR [21]. Using a synthetic metagenomic sample with a perfect ground truth of almost all contigs [18], we evaluated the retrieval performance of available normalization methods and clustering algorithms from the HiCBin and other Hi-C-based analysis pipelines and assessed the impacts of spurious contact detection step on the binning results. Finally, we compared the MAG retrieval ability of HiCBin against all the other state-of-the-art Hi-C-based binning pipelines: ProxiMeta, bin3C, and MetaTOR [20–22], and one widely used the shotgun-based binning software MetaBAT2 [33] on a human gut dataset [20] and a wastewater dataset [28].

## Results

### Analyses of the metagenomic yeast sample

A total of 6566 contigs longer than 1000 bp were assembled with the total length of 126,030,343 bp. There were 4,700,202 Hi-C read pairs subsequently aligned to different contigs. Among the contigs, 5283 of them passed the filtering criteria and represented 80.5% of all assembled contigs and 98.6% of the total length of the entire shotgun assembly. For the normalization step, 2700 contigs were labeled by TAXAassign, generating 847,109 intra-species contacts to fit the HiCzin model. We discarded normalized contacts below 0.178 as spurious contacts. Thereafter, 14 valid genome bins with the bin size larger than 150 kbp were obtained by the Leiden algorithm with a resolution parameter as 1. These valid bins contained 5217 contigs with the total length of 124,120,326 bp, representing 98.5% of the total length of all shotgun assembled contigs longer than 1000 bp. The *F*-score, ARI, and NMI were 0.908, 0.894, and 0.895, respectively. We then investigated the influences on the binning performance of available normalization methods and clustering algorithms from the HiCBin and other metagenomic Hi-C analysis pipelines and evaluated the impacts of spurious contact detection step on the binning results based on our gold standards.

#### HiCzin normalization outperforms other normalization methods on binning

Apart from a state-of-the-art normalization method HiCzin [27], several relatively simple metagenomic Hi-C normalization methods have been developed. Beitel et al. [19] divided raw Hi-C contacts by the product of the length of two contigs. MetaTOR [21] normalized raw Hi-C interactions by the geometric mean of the contigs’ coverage. Metaphase [18] and bin3C [22] divided raw Hi-C counts by the product of the number of restriction sites, and bin3C used the Knight-Ruiz algorithm [24] to construct a general doubly stochastic matrix after the first step correction. For convenience, we denote those normalization methods by site, length, and coverage as naive site, naive length, and naive coverage and denote the two-stage normalization method in bin3C as bin3C_Norm. We normalized the raw Hi-C contacts by different normalization methods and applied the Leiden algorithm with the same resolution parameter to cluster the contigs. The gold standards were utilized to evaluate the binning results. As shown in Table 1, HiCzin achieved the best binning performance among all five normalization methods.

**Table 1:** HiCzin normalization followed by the Leiden clustering algorithm outperforms other normalization methods on binning in terms of F-score, ARI, and NMI

	No. of groups	No. of contigs	*F*-score	ARI	NMI
Naive site	13	5191	0.873	0.854	0.865
Naive length	13	5210	0.892	0.876	0.873
Naive coverage	11	5240	0.761	0.702	0.847
bin3C_Norm	13	5125	0.894	0.878	0.884
HiCzin (HiCBin)	14	5217	**0.908**	**0.894**	**0.895**

*Note*: the optimal values of the results are in bold. No. of groups represents the number of valid genome bins, and No.# of contigs is the number of contigs in the valid bins

#### The Leiden community detection algorithm outperforms other clustering methods

Besides the Louvain algorithm and the Leiden algorithm (see the “Methods” section), there are several widely-used network clustering algorithms, such as the Markov clustering algorithm [34], Infomap algorithm [25], and label propagation algorithm [35]. Markov clustering and Infomap algorithm are both based on flow models and have already been employed to cluster contigs using metagenomic Hi-C contact maps [19, 22]. The label propagation algorithm iteratively repeats a process where each node in the graph adopts the most common label among its neighbors. Some studies have already compared part of these community detection algorithms in the benchmarking networks [36, 37]. However, none of these comparisons focused on the metagenomic Hi-C data. In addition, as one of the latest network clustering algorithms, the Leiden algorithm was seldomly taken into consideration for comparison. Therefore, we investigated the performance of different community detection algorithms in clustering metagenomic Hi-C contact graph. We applied multiple algorithms in the clustering step of the HiCBin pipeline and evaluated the valid genome bins generated by different clustering algorithms (Table 2). Both flow-based algorithms generated much more communities than the real number of species with poor clustering quality. The label propagation algorithm could not detect all species. Two modularity-based algorithms (i.e., the Louvain algorithm and the Leiden algorithm) obtained better performance than other algorithms. The Leiden algorithm provided the best binning results and had a significant improvement to the Louvain algorithm.

**Table 2:** The Leiden algorithm outperforms other clustering methods including label propagation, Markov clustering, Infomap, and Louvain for Hi-C data analysis based on the contacts normalized by HiCzin

	No. of groups	No. of contigs	*F*-score	ARI	NMI
Label propagation	10	5026	0.680	0.599	0.739
Markov clustering	37	4699	0.546	0.471	0.686
Infomap	65	3582	0.772	0.731	0.770
Louvain	14	5241	0.838	0.815	0.842
Leiden (HiCBin)	14	5217	**0.908**	**0.894**	**0.895**

*Note*: The optimal values of the results are in bold. No. of groups represents the number of valid genome bins. No. of contigs is the number of contigs in the valid bins

#### Spurious contact detection improves the performance of HiCBin

As spurious contact detection is a novel step in metagenomic Hi-C binning pipelines, we explored how this new step benefits the HiCBin pipeline. By discarding normalized contacts below 0.178, we could remove 46.9% of spurious contacts while only 5.6% of valid contacts were incorrectly discarded, which was close to our preselected percentage of acceptable incorrectly identified valid contacts in the whole data. Thus, a large fraction of spurious contacts were removed while most of the valid contacts were retained. Moreover, the HiCBin pipeline was also run without the step of spurious contact detection, where the *F*-score, ARI, and NMI were 0.904, 0.890, and 0.891, respectively. These three standards were increased to 0.908, 0.894, and 0.895 after removing spurious contacts. Therefore, spurious contact detection indeed improved the binning performance.

#### HiCBin outperforms other Hi-C-based binning methods

We compared the binning performance of HiCBin to two publicly available metagenomic binning pipelines, MetaBAT2 and bin3C, on the metagenomic yeast dataset (Table 3). MetaBAT2 is a conventional shotgun-based binning pipeline, and bin3C is an open-source solutions to deconvolute the metagenomic Hi-C datasets (see the “Methods” section). Noticeably, as a publicly available Hi-C-based binning tool, MetaTOR splits contigs into “chunks” of nearly fixed size after the assembly and attempts to bin these chunks instead of the assembled contigs. It is not valid to compare MetaTOR to other pipelines without splitting based on our gold standards due to different clustering objectives. Therefore, we did not include MetaTOR within the comparison for the metagenomic yeast dataset.

**Table 3:** Comparison of the performance of Hi-C-based binning pipelines: HiCBin and bin3C, and a shotgun-based binning tool MetaBAT2 based on all contigs

	No. of groups	No. of contigs	*F*-score	ARI	NMI
MetaBAT2	14	2827	0.607	0.478	0.705
bin3C	84	3348	0.651	0.576	0.726
HiCBin	14	5217	**0.908**	**0.894**	**0.895**

*Note*: The optimal values of the results are in bold. No. of groups represents the number of valid genome bins. No. of contigs is the number of contigs in the valid bins

Without using Hi-C information, MetaBAT2 only binned fewer than half of the contigs with relatively poor quality. bin3C had better binning results than MetaBAT2. However, the number of communities generated by bin3C was much larger than the real number of species, which was consistent with the result of the Infomap algorithm as bin3C utilized the Infomap algorithm to do clustering. HiCBin included almost all contigs in the valid genome bins and achieved much better binning performance than all other comparable binning pipelines on the metagenomic yeast dataset.

Moreover, we evaluated the clustering performance of contigs that were not annotated by TAXAassign (Table 4). HiCBin still outperformed both shotgun-based and Hi-C-based binning tools for contigs without any prior information, which indicated that the improved binning performance was due to better algorithms of HiCBin for analyzing metagenomic Hi-C data.

**Table 4:** Comparison of the performance of Hi-C-based binning pipelines: HiCBin and bin3C, and a shotgun-based binning tool MetaBAT2 based on contigs that were not annotated by TAXAassign

	*F*-score	ARI	NMI
MetaBAT2	0.639	0.512	0.675
bin3C	0.670	0.581	0.691
HiCBin	**0.935**	**0.924**	**0.890**

*Note*: The optimal values of the results are in bold

### Analyses of the human gut sample

We assembled 105,267 contigs longer than 1000 bp with a total length of 530,969,816 bp. Two Hi-C libraries were merged, and a total of 11,633,561 Hi-C sequencing read pairs were mapped mates to different contigs. There were 66,809 contigs totaling 457,763,358 bp in length that passed the contig filtering restriction and accounted for 86.2% of the total length of all assembled contigs. In the normalization step, TAXAassign labeled 6225 contigs and generated 850,289 intra-species contact samples to fit the HiCzin model. Normalized contacts below 0.248 were discarded as spurious contacts. The resolution parameter was tuned to be 20 for clustering. After binning from contact maps, 194 valid genome bins were identified with a total size of 434,186,687 bp. Among these bins, 36 valid bins totaling 180,682,506 bp in size were partially contaminated and were subsequently post-processed, generating 180 sub-bins with a total bin size of 148,154,265 bp. Therefore, HiCBin recovered 338 valid genome bins in total. These 338 bins ranging from 150,395 to 4,884,489 bp had a total size of 401,658,446 bp and represented 75.6% of the total length of the whole assembled contigs.

**Fig. 1:**
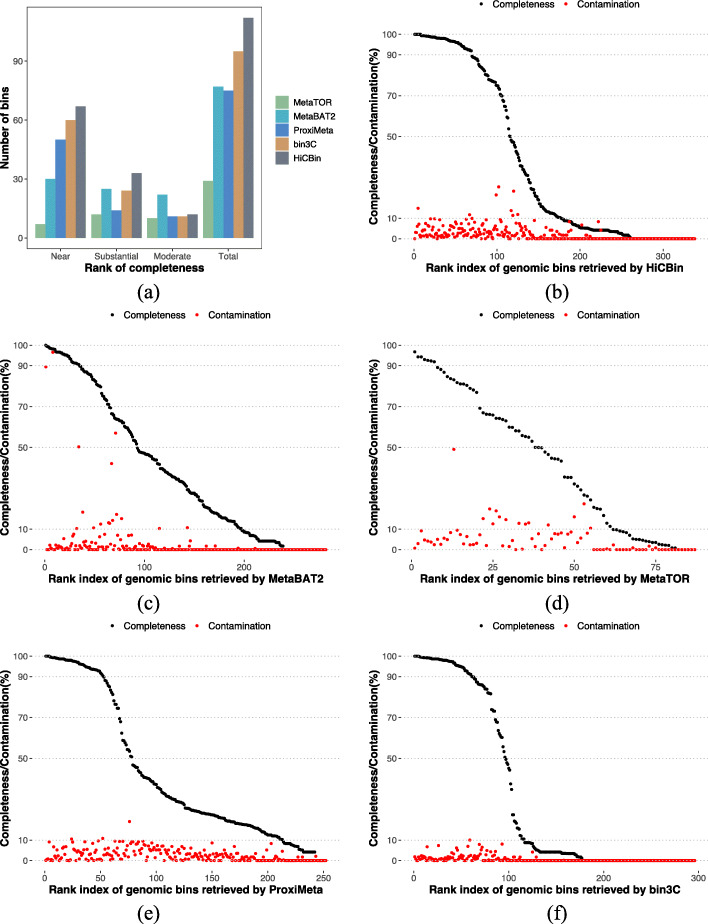
HiCBin outperforms other binning methods on the human gut dataset. **a** Comparison of high-quality draft genomic bins retrieved by different binning pipelines on the human gut dataset according to the CheckM rank for completeness (near-complete: ≥ 90% completeness, ≤ 10% contamination; substantially complete: ≥ 70% and <90*%* completeness, ≤ 10% contamination; moderately complete: ≥ 50% and <70*%* completeness, ≤ 10% contamination). Completeness (black) and contamination (red) of the draft genomic bins retrieved by **b** HiCBin, **c** MetaBAT2, **d** MetaTOR, **e** ProxiMeta, and **f** bin3C on the human gut dataset. The *x*-axis shows the ranks of bins according to their completeness

We compared the MAG retrieval ability of HiCBin to all the other Hi-C-based binning pipelines (ProxiMeta, bin3C, and MetaTOR) and one conventional shotgun-based binning tool (MetaBAT2) according to the CheckM standard for high-quality draft genomic bins (Fig. 1a). The CheckM validation results of ProxiMeta and bin3C came from the supporting materials of their papers [20, 22]. HiCBin retrieved 67 near-complete, 33 substantially complete, and 12 moderately complete MAGs (Fig. 1b, Additional file 2). Near-complete MAGs ranged from 1,509,376 bp to 4,884,489 Mbp, while the substantially complete MAGs ranged from 1,549,630 to 2,662,928 bp, and moderately complete MAGs ranged from 1,336,581 to 2,881,511 bp. In comparison, MetaBAT2 generated 30 near-complete, 25 substantially complete, and 22 moderately complete MAGs (Fig. 1c, Additional file 3). Genomic bins recovered by MetaTOR suffered serious contaminations without the recursive Louvain clustering. The average contamination of bins with completeness larger than 50% was 80.94%. One potential explanation for the high contamination of draft genomes was that the Louvain algorithm in MetaTOR utilized the Newman-Girvan criterion as the modularity function, which could not identify small genomes due to the resolution limit in the complex Hi-C contact network with a large number of contigs, and those unidentified small genomes were merged into other draft genomes. After the recursive binning procedure, the average contamination of bins with completeness above 50% was decreased to 7.8% and 7 near-complete, 12 substantially complete, and 10 moderately complete MAGs were retrieved by MetaTOR (Fig. 1d, Additional file 4). ProxiMeta created 247 bins with 50 near-complete, 14 substantially complete, and 11 moderately complete MAGs (Fig. 1e) while bin3C constructed 138 bins with 60 near-complete, 24 substantially complete, and 11 moderately complete MAGs (Fig. 1f). These two Hi-C-based binning pipelines had better binning results than MetaBAT2, demonstrating the high resolution of Hi-C data. HiCBin achieved the best performance in resolving high-quality MAGs on the human gut dataset. Moreover, it was reported in the bin3C paper that contigs totaling 290,643,239 bp and representing 40.4% of the total length of the assembly were included in the bins larger than 50 kbp. Even though HiCBin selected a stricter requirement on minimum bin size, our pipeline still improved the total length of contigs in bins by 38%. As for the low-quality draft genomic bins retrieved by different binning methods (Additional file 1: Fig. S1), ProxiMeta could construct the most weak-complete draft genomes while bin3C generated the fewest contaminated bins.

Besides the CheckM criteria, we also validated our method using the contigs annotated by TAXAassign in terms of *F*-score, ARI, and NMI to consolidate our results (see the “Methods” section). Among 6225 labeled contigs, 3113 contigs were randomly selected out as the ground truth to do the evaluation while the remaining 3112 contigs served as the input of HiCBin. Since MetaTOR splits the long contigs into shorter pieces referred to as chunks and then clusters these chunks while other binning methods bin the assembled contigs, it is not valid to compare MetaTOR to other binning pipelines without splitting in terms of *F*-score, ARI, and NMI. Moreover, ProxiMeta is proprietary without the open-source software. Therefore, we compared HiCBin to the remaining two binning tools, i.e., one shotgun-based binning method MetaBAT2 and one Hi-C-based binning pipeline bin3C (Table 5). HiCBin still outperformed both binning methods, which was consistent with the CheckM validation.

**Table 5:** Comparison of the binnig performance of MetaBAT2, bin3C, and HiCBin using half of the contigs that were labeled by TAXAassign in terms of *F*-score, ARI, and NMI on the human gut sample

	*F*-score	ARI	NMI
MetaBAT2	0.748 (0.010)	0.724 (0.011)	0.779 (0.003)
bin3C	0.721 (0.014)	0.705 (0.014)	0.775 (0.004)
HiCBin	**0.816** (0.013)	**0.800** (0.015)	**0.825** (0.005)

*Note*: The optimal values of the results are in bold. Values in the parentheses represent the standard deviations of ten times validation experiments

Finally, we explored the impacts of spurious contact detection on this real microbiome community sample. Without spurious contact detection, the HiCBin pipeline could retrieve 63 near-complete and 24 substantially complete MAGs, which were improved to 67 near-complete and 33 substantially complete MAGs after removing spurious contacts, indicating the spurious contact detection step could improve the binning quality.

### Analyses of the wastewater dataset

As the wastewater microbiome community sample was processed by the proprietary Hi-C preparation kit, the restriction enzymes utilized in the experiment were not specified, resulting in the lack of information on the number of restriction sites for contigs. Therefore, HiCzin_LC mode was employed for normalization [27]. Moreover, compared to the human gut dataset, the wastewater dataset was much more complicated with 752,580 assembled contigs longer than 1000 bp. The total length of the whole assembled contigs was 1,910,562,642 bp. A total of 22,277,042 Hi-C read pairs were aligned to different contigs. There were 493,944 contigs that satisfied the filtering restrictions. These contigs totaled 1,519,539,584 bp in length, representing 79.5% of the total length of the whole assembled contigs longer than 1000 bp. For the normalization step, 9,745,145 intra-species contact samples were generated by TAXAassign to fit the HiCzin_LC model, and then, we discarded normalized contacts below 0.625 as spurious contacts. After tuning the hyper-parameter, contigs were clustered by the Leiden algorithm with resolution parameter as 20. A total of 391 valid genome bins were identified with the total size of 1,505,330,057 bp. Among them, 152 bins were partially contaminated. After the post-processing step, 1014 sub-bins were generated with a total bin size of 963,363,324 bp. In total, HiCBin reconstructed 1253 valid genome bins with a total size of 1,434,645,324 bp, ranging from 150,008 to 8,885,313 bp and accounting for almost 75.1% of the total length of overall shotgun contigs.

**Fig. 2:**
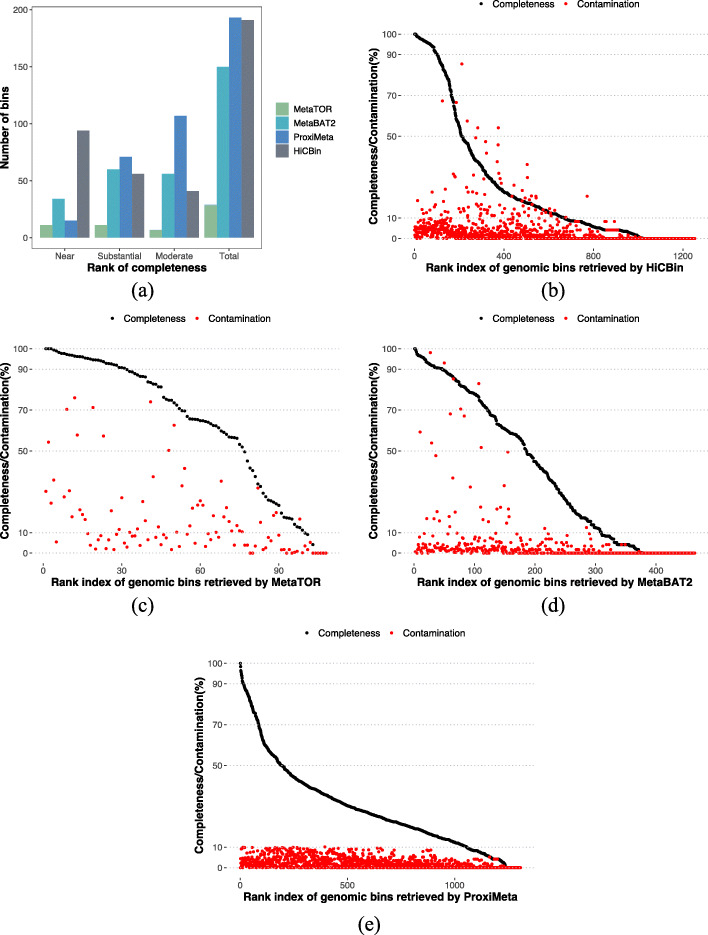
HiCBin outperforms other binning methods on the wastewater dataset. **a** Comparison of high-quality draft genomic bins retrieved by different binning pipelines on the wastewater dataset according to the CheckM rank for completeness (near-complete: ≥ 90% completeness, ≤ 10% contamination; substantially complete: ≥ 70%, <90*%* completeness, ≤ 10% contamination; moderately complete: ≥ 50%, <70*%* completeness, ≤ 10% contamination). Completeness (black) and contamination (red) of the draft genomic bins retrieved by **b** HiCBin, **c** MetaTOR, **d** MetaBAT2, and **e** ProxiMeta on the wastewater dataset. The *x*-axis shows the ranks of bins according to their completeness

According to the CheckM completeness standard for high-quality draft genomic bins, HiCBin achieved the best binning results on the wastewater dataset compared to other binning pipelines (Fig. 2a). Specifically, HiCBin retrieved 94 near-complete, 56 substantially complete, and 41 moderately complete MAGs (Fig. 2b, Additional file 5). Near-complete MAGs ranged from 977,957 to 5,330,262 bp, while the substantially complete MAGs ranged from 751,056 to 5,316,909 bp and moderately complete MAGs ranged from 780,925 to 8,885,313 bp. Noticeably, bin3C required the input of the names of restriction enzymes as it normalized the raw Hi-C contacts using the number of restriction sites on contigs. Since the enzymes utilized in the experiment were unknown, bin3C was not applicable to this dataset. MetaTOR recovered 11 near-complete, 11 substantially complete, and 7 moderately complete MAGs (Fig.2c, Additional file 6) while MetaBAT2 retrieved 34 near-complete, 60 substantially complete, and 56 moderately complete MAGs (Fig. 2d, Additional file 7). ProxiMeta generated 1288 bins with 15 near-complete, 71 substantially complete, and 107 moderately complete MAGs (Fig. 2e). Although the total number of high-quality MAGs retrieved by ProxiMeta was similar to that of HiCBin, HiCBin generated 94 near-complete MAGs, a gain of 527% against the result of ProxiMeta, indicating that the quality of MAGs retrieved by HiCBin was much higher than ProxiMeta. As for the low-quality draft genomic bins constructed by different binning tools (Additional file 1: Fig. S2), ProxiMeta generated the most weak-complete draft genomes, followed by HiCBin. The number of weak-complete bins retrieved by ProxiMeta and HiCBin was much higher than that retrieved by the other two binning pipelines.

We also evaluated the binning performance of HiCBin using labeled contigs provided by TAXAassign in terms of *F*-score, ARI, and NMI (see the “Methods” section). We randomly selected 17,481 labeled contigs from a total of 34,961 contigs annotated by TAXAassign and these contigs served as the ground truth for the binning evaluation. The remaining half of labeled contigs were utilized to generate the intra-species Hi-C interactions in the normalization step and determine the resolution parameter in the clustering step by HiCBin. The binning performance of MetaTOR and ProxiMeta could not be evaluated by this method due to the same reasons explained in the subsection of analyzing the human gut sample. Therefore, we evaluated the bins retrieved by HiCBin and MetaBAT2 (Table 6) and HiCBin outperforms MetaBAT2 in terms of *F*-score, ARI and NMI.

**Table 6:** Comparison of the binnig performance of MetaBAT2 and HiCBin using half of the contigs that were labeled by TAXAassign in terms of *F*-score, ARI, and NMI on the wastewater sample

	*F*-score	ARI	NMI
MetaBAT2	0.593 (0.009)	0.576 (0.009)	0.830 (0.003)
HiCBin	**0.724** (0.005)	**0.710** (0.006)	**0.873** (0.002)

*Note*: The optimal values of the results are in bold. Values in the parentheses represent the standard deviations of ten times validation experiments

### Running time of HiCBin, MetaBAT2, and bin3C

HiCBin, bin3C, and MetaBAT2 only contain the binning analysis, whereas MetaTOR uses its own workflow and integrates the binning process with standard alignment and annotation softwares. Therefore, it is reasonable to compare the running time of binning directly among HiCBin, MetaBAT2, and bin3C. All pipelines were executed on one computing node of a 2.40-GHz Intel Xeon Processor E5-2665 provided by the Advanced Research Computing platform at University of Southern California. A 50,000-MB memory was allocated to the computing node. The running time of HiCBin and MetaBAT2 on three datasets is shown in Table 7. bin3C was only run on the metagenomic yeast dataset and consumed 21 min and 16 s, which is slower than HiCBin. Though both Hi-C-based binning pipelines perform better than the shotgun-based binning tool MetaBAT2, MetaBAT2 runs faster than HiCBin and bin3C on the metagenomic yeast dataset. HiCBin consumes less time than MetaBAT2 on the human gut dataset but consumes more time than MetaBAT2 on the wastewater dataset. This is because it takes relatively long time for HiCBin to generate the observations of the intra-species contacts in the normalization step for the datasets with a huge number of contigs.

**Table 7:** Running time of HiCBin and MetaBAT2 on the yeast, human gut, and wastewater datasets

	Metagenomic yeast	Human gut	Wastewater
HiCBin	14 min 50 s	54 min 33 s	58 h 7 min
MetaBAT2	1 min 52 s	1 h 39 min	12 h 34 min

Moreover, compared to the standard Hi-C-based analysis pipeline, HiCBin requires an extra preprocessing step on running the TAXAassign software in order to annotate some contigs. The TAXAassign software was run on a machine with 4-way 6-core 1.87-GHz Intel Xeon CPUs and spent 6.5 h, 7.5 h, and 17.7 h annotating contigs assembled from the synthetic yeast, human gut, and wastewater datasets, respectively.

## Discussion

We have introduced a new open-source tool HiCBin to resolve high-quality MAGs using Hi-C contact maps. The use of the metagenomic yeast sample with a perfect ground truth of contigs’ species identity allowed us to evaluate the different normalization methods and clustering algorithms utilized in the HiCBin and other available metagenomic Hi-C analysis pipelines. We found that the HiCzin normalization method and the Leiden algorithm combined with the Potts spin-glass model from the HiCBin provided the best results in genome binning. The spurious contact detection step proved useful in contig binning on both the metagenomic yeast dataset and the human gut dataset. Finally, we validated that HiCBin was the best approach to the accurate retrieval of MAGs on the human gut dataset and wastewater dataset compared to all the other Hi-C-based binning pipelines and one traditional shotgun-based software. HiCBin even achieved more than five times improvement against the previous best result in recovering near-complete MAGs for the wastewater dataset. Moreover, HiCBin could include more than 75% of the total length of the whole assembled contigs into bins larger than 150 kbp for both real microbial community samples, representing a marked improvement in the metagenomic binning domain. Notably, the performance of the Hi-C-based binning pipelines is superior to the traditional approach in most instances, indicating the great potential of metagenomic Hi-C data.

As the restriction enzymes utilized in Hi-C experiments are unspecified under some circumstances, those pipelines using the information of the number of restriction sites to do normalization cannot be executed. Employing the HiCzin mode that merely normalized raw Hi-C contact maps by the length and coverage of contigs rendered the HiCBin pipeline more applicable.

From our observation, the selection of minimum bin size is relatively stable. Though different binning pipelines may choose different bin size thresholds, we found that the size of high-quality MAGs determined by CheckM is always much larger than those minimum bin size thresholds.

Though HiCBin requires an extra preprocessing step on running the TAXAassign software and thus takes extra time compared to the standard Hi-C-based analysis pipeline, the time consumed by TAXAassign is acceptable, especially when compared to the time needed for the assembly of long contigs from short reads, which always requires much more time on real metagenomic datasets.

Although HiCBin performed well in the analysis of Hi-C data, it was not designed for other proximity-ligation techniques such as meta3C [23]. The whole analyses of the metagenomic yeast sample in this paper were at the species level as we found it challenging to annotate contigs at the strain level. In the future, it is necessary to explore the contig binning at the strain level. For the two real microbial community datasets, we employed the CheckM as one method to evaluate the binning performance. Though CheckM is the most popular method to evaluate the quality of bins on real samples, how accurately the marker-gene based validation could reflect the true completeness and contamination of recovered MAGs deserves further exploration considering that some genomic regions do not contain marker genes.

## Conclusions

HiCBin can accurately bin metagenomic contigs and retrieve high-quality MAGs using Hi-C contact maps. The complete draft genomes directly recovered from microbial communities can significantly facilitate the downstream analyses, such as tracking horizontal gene transfer and probing virus-host interactions.

## Methods

### Datasets

We analyzed the performance of our genome binning tool HiCBin on three published metagenomic Hi-C datasets [18, 20, 28].

#### Synthetic metagenomic yeast community

The synthetic metagenomic yeast (M-Y) sample consist of 16 yeast strains from 13 yeast species (BioProject: PRJNA245328, Accession: SRR1263009 and SRR1262938) [18]. The Nextera DNA Sample Preparation Kit (Illumina) was employed to prepare the shotgun library (SRR1262938). Hi-C library (SRR1263009) was created using HindIII and NcoI restriction endonuclease (NEB). Paired-end sequencing of Hi-C reads was performed using the HiSeq and MiSeq Illumina platforms. The raw WGS dataset contains 85.7 million read pairs (101 bp per read) and the raw Hi-C dataset includes 81 million read pairs (100 bp per read). As the reference genomes of all strains in the metagenomic sample are known, we can determine the true species identity of the assembled contigs by aligning assembled contigs to reference genomes at the species level and then construct a gold standard to validate the whole-community genome binning performance (described below).

#### Real microbiome communities from a human gut sample and a wastewater sample

To compare HiCBin to a proprietary metagenome genome binning software (ProxiMeta), two publicly available metagenomic Hi-C sequencing datasets constructed by the ProxiMeta Hi-C kit (Phase Genomics, Seattle, WA, USA) were chosen [20, 28].

The first dataset was derived from a fecal sample of a human subject (BioProject: PRJNA413092, Accession: SRR6131122, SRR6131123, and SRR6131124) [20]. Two four-cutter restriction enzymes MluCI and Sau3AI (New England Biolabs) were utilized to construct two different Hi-C libraries (SRR6131122, SRR6131124). The shotgun and Hi-C libraries were sequenced by a Illumina HiSeqX platform at 151 bp. The raw shotgun library (SRR6131123) consisted of 250,884,672 read pairs while the sequencing of two Hi-C libraries produced 41,733,770 read pairs (Sau3AI library) and 48,798,091 read pairs (MluCI library), respectively.

The second dataset was generated from a wastewater (WW) sample (BioProject: PRJNA506462, Accession: SRR8239392 and SRR8239393) [28]. The shotgun library (SRR8239393) and Hi-C library (SRR8239392) were prepared using the DNeasy PowerWater kit and ProxiMeta Hi-C kit, respectively. After sequencing, both libraries by HiSeq 4000 at 150 bp, 269,312,499 and 95,284,717 read pairs for the wastewater shotgun and Hi-C libraries were produced, respectively. Noticeably, the restriction enzymes employed in the experiment were unspecified due to the proprietary Hi-C preparation kit.

### Preprocessing raw reads

A standard cleaning procedure was applied to all raw WGS and Hi-C read libraries using bbduk from the BBTools suite (v37.25) [38]. We discarded short reads below 50 bp at each cleaning step. Adaptor sequences were removed by bbduk with parameter “ktrim=r k=23 mink=11 hdist=1 minlen=50 tpe tbo” and reads were quality-trimmed using bbduk with parameters “trimq=10 qtrim=r ftm=5 minlen=50.” Then, the first 10 nucleotides of each read were trimmed by bbduk with parameter “ftl=10.”

### Shotgun assembly

De novo metagenome assembly was produced by MEGAHIT (v1.2.9) [39] with parameters “-min-contig-len 300 -k-min 21 -k-max 141 -k-step 12 -merge-level 20,0.95” using processed shotgun reads and contigs below 1 kb were discarded (Table 8).

**Table 8:** Statistics of assembled contigs for three datasets

Dataset	Contigs ≥ 1 kbp	N50	Average length	Total length
Metagenomic yeast	6566	63,305	10,781	126,030,343
Human gut	105,267	14,166	5044	530,969,816
Wastewater	752,580	2977	2538	1,910,562,642

### Hi-C read alignment

For the Hi-C library, only paired reads were retained for the downstream workflow. All identical PCR optical and tile-edge duplicates for Hi-C paired-end reads were removed by the script “clumpify.sh” from BBTools suite (v37.25) [38] with default parameters. Then, processed Hi-C paired-end reads were aligned to assembled contigs using BWA MEM (v0.7.17) [40] with parameter “-5SP.” Samtools (v1.9) [41] with parameters “view -F 0x904” were subsequently applied to the resulting BAM files to remove unmapped reads, secondary alignments, and supplementary alignments. Alignments with low quality (nucleotide match length < 30 or mapping score < 30) were filtered out.

### Generating Hi-C contact maps

Raw contig-contig interactions were aggregated as contacts by counting the number of alignments bridging two contigs [42]. As contacts reflect the proximity extent between contigs, only pairs of Hi-C reads aligned to different contigs were retained to generate the contact maps. We then denote the contig signal as the number of Hi-C reads mapped to the contig. In the Hi-C experiment, shorter contigs with smaller signals tend to have much higher variance, weakening the normalization and clustering stability in the downstream analysis. To get rid of these deleterious impacts, restrictions on minimum contig length (default, 1000 bp) and minimum contig signal (default, two) were imposed to filter problematic contigs. We discarded contigs failing to satisfy either of the two limitations. In this way, raw contact maps were generated from the Hi-C read pairs to measure the interactions between contigs.

### Normalizing the raw contact map

Apart from chromosomal contacts of interest, two types of experimental biases have been reported to have substantial effects on raw metagenomic Hi-C contact maps, rendering the normalization of Hi-C contact maps essential for the binning [27]. Therefore, we employed HiCzin, a state-of-the-art metagenomic Hi-C normalization method, to remove the influences of biases [27]. We denote the intra-species contacts as the count of Hi-C interactions between contigs from the same species and refer to the non-zero intra-species contacts as valid contacts. Based on the assumption that the observed intra-species contacts follow zero-inflated negative binomial distribution, the HiCzin model corrected biases of the number of restriction sites, length, and coverage of contigs while taking the biases of unobserved Hi-C interactions into account. Considering the restriction enzymes utilized in Hi-C experiments that are unspecified in some real situations, HiCzin also developed a special mode (HiCzin_LC) that merely normalized raw Hi-C contact maps by the length and coverage of contigs. The main procedure of the HiCzin normalization contained the following steps.

#### Computing the coverage of assembled contigs

The coverage of contigs was computed using MetaBAT2 (v2.12.1) [12, 33] script: “jgi_summarize_bam_contig_depths.”

#### Generating the intra-species contacts

To generate the observations of the intra-species contacts, TAXAassign (v0.4) (https://github.com/umerijaz/TAXAassign) [43] was utilized to resolve the taxonomic assignment of contigs using NCBI’s Taxonomy and its nt database with parameters “-p -c 20 -r 10 -m 98 - q 98 -t 95 -a “0,70,80,95,95,98” -f.” We discarded indeterministic assignment results with “unclassified” labels at the species level. In this way, some contigs could be unambiguously annotated at the species level. Intra-species pairs were subsequently constructed by pairwise combining contigs assigned to the same species and corresponding contacts were regarded as the samples of the intra-species contacts.

#### Fitting the regression model

All intra-species contact samples were utilized to fit the HiCzin or HiCzin_LC model, combining the negative binomial distribution of the intra-species contacts with a mass distribution of unobserved contacts. The residues of the counting part served as the normalized contacts.

### Removing spurious contacts

Based on the expectation that the magnitude of the normalized spurious contacts by HiCzin or HiCzin_LC to be significantly smaller than that of the normalized valid contacts [27], we discarded the normalized contacts below a threshold as spurious contacts, and determined the threshold such that less than a preselected percentage (default, 5%) of non-zero intra-species contact samples generated by TAXAassign are incorrectly identified as spurious contacts. This percentage reflected the acceptable fraction of losses of the valid contacts in the whole data. In this way, we could keep the proportion of incorrectly discarded intra-species contacts under control while discarding most of the spurious contacts as shown in previous work [27].

### Clustering by the Leiden algorithm

Compared to classical clustering algorithms [44], such as K-means, K-medoids, and Gaussian mixture model, community detection algorithms do not require the input of the number of clusters, which is extremely challenging to determine [45]. The Louvain algorithm is one of the most popular community detection algorithms used to cluster contigs based on metagenomic Hi-C data [21, 30, 46]. As a hierarchical agglomerative method, the Louvain algorithm takes a two-stage greedy approach to optimizing the modularity function [47]. Specifically, the algorithm iterates and assigns each node to a community such that the local movement will increase the modularity function, followed by aggregating the network. This process repeats iteratively until convergence. However, many recent experiments have shown that such kind of local movement may identify disconnected communities within groups [29], weakening the interpretability of the MAG retrieval. To address this problem, we introduced the Leiden algorithm [29], one of the most novel and advanced community detection algorithms, into the metagenomic Hi-C binning domain. The Leiden algorithm is also a modularity-based algorithm and improves the Louvain algorithm by refining the partition before aggregating the network to guarantee the connectivity of each community. Additionally, the Leiden algorithm is much faster than the Louvain algorithm by a fast local move approach [29]. In practice, the Leiden algorithm has achieved good performance in clustering transcriptomics data [48] and gene expression data [49]. Thus, we applied this state-of-the-art clustering algorithm to the normalized Hi-C contact maps.

Selecting the objective function is crucial for modularity-based algorithms. The classical Newman-Girvan criterion [47] is restricted by a resolution limit and may fail to identify small communities [32]. To overcome the resolution limit in the modularity context, we selected a general and flexible measure of community structure based on the Reichardt and Bornholdt’s Potts model [31] as the modularity function of the Leiden algorithm, i.e.:

1 $$ \begin{aligned} Q = \sum_{ij}\left(A_{ij} - \gamma \frac{k_{i} k_{j}}{2m}\right)\delta\left(c_{i}, c_{j}\right), \end{aligned}  $$

where *Q* is the modularity function, *A* is the adjacency matrix, *k*_*i*_ is the weighted degree of contig *i*, *m* is the total edge weight, *γ*>0 is a resolution parameter, *c*_*i*_ denotes the community of contig *i*, and *δ*(*c*_*i*_,*c*_*j*_)=1 if *c*_*i*_=*c*_*j*_ and 0 otherwise. The resolution parameter *γ* in front of the configuration null part controls the relative importance between links within the communities and the null model. Noticeably, this hyperparameter determines the number of communities. Higher resolutions always lead to more communities. Therefore, tuning this parameter is important for the clustering algorithm.

As a few contigs have been unambiguously annotated by TAXAassign in the normalization step, we took advantage of these taxonomic assignment results to select an optimal resolution parameter. For each candidate resolution parameter chosen from 1, 5, 10, 15, 20, 25, and 30, we clustered the whole set of contigs and then computed two effective clustering evaluation measures (Additional file 1): Adjusted Rand Index (ARI) and normalized mutual information (NMI) utilizing those contigs that could be labeled by TAXAassign. We calculated the resolution score as the average of these two measures and selected the candidate producing the highest resolution score as the optimal value of the hyperparameter.

After determining the resolution parameter, we could finally cluster the whole set of contigs. We set the minimum bin size as default 150 kbp, which was slightly smaller than the minimal length of known bacterial genomes [50]. Only contig bins above 150 kbp were regarded as valid genome bins for the downstream workflow.

### Gold standards to evaluate binning performance for the M-Y sample

**Fig. 3:**
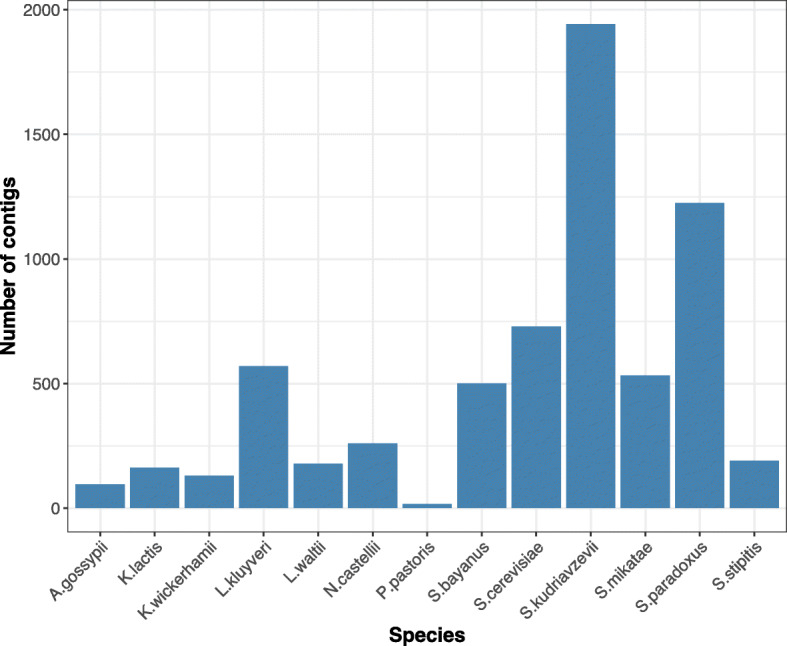
The number of assembled contigs for each of the 13 species in the metagenomic yeast sample

To evaluate the contig binning results for the metagenomic yeast sample, a perfect ground truth for the species identity of almost all assembled contigs was constructed. We first downloaded the reference genomes of all 16 yeast strains in the metagenomic sample (Additional file 1: Table S1) [18]. As the analyses were made at the species level, genomes of four strains (FY, CEN.PK, RM11-1A, and SK1) from the same species (*Saccharomyces cerevisiae*) were merged into one reference genome. Then, assembled contigs were aligned to those 13 reference genomes of all known species by BLASTN [51] with parameters: “-perc identity 95 -evalue 1e-30 -word size 50.” The true species identity of the assembled contigs could be determined if there existed any alignment of the contigs to the species’ reference genome (Fig. 3). In this way, 6529 (99.6%) out of a total of 6566 contigs could be aligned to reference genomes. After we obtained the true labels of contigs, we employed three comprehensive clustering performance metrics (Additional file 1): Fowlkes-Mallows scores (*F*-scores), Adjusted Rand Index (ARI), and normalized mutual information (NMI). These three metrics served as the gold standards to evaluate binning performance for the M-Y sample.

### Analyzing genome bins for the human and wastewater samples

For the human gut and wastewater datasets, as we did not know the ground truth of most of the contig identities, we employed two methods to evaluate the binning performance.

We firstly employed CheckM [52] (v1.1.3, parameter: lineage_wf), which searched the single-copy marker genes in the bins, to evaluate draft assembled genomes. According to the CheckM criteria for completeness [20], high-quality draft genomes could be assigned to three ranks, i.e., near-complete (≥ 90% completeness, ≤ 10% contamination), substantially complete (≥ 70% and < 90*%* completeness, ≤ 10% contamination), and moderately complete (≥ 50% and <70*%* completeness, ≤ 10% contamination). We also defined two categories of low-quality draft genomes, i.e., weak-complete (≥ 20% and <50*%* completeness, ≤ 10% contamination) and contaminated (≥ 20% completeness, > 10% contamination).

To consolidate our validations on real samples, we designed the second evaluation method using the contigs annotated by TAXAassign in the normalization step. Specifically, we randomly divided the labeled contigs into two equal parts. One part was utilized to construct the intra-species contacts and determine the resolution parameter by HiCBin while the other part served as the ground truth to evaluate the binning performance of different software packages in terms of *F*-score, ARI, and NMI. The validation experiments were repeated ten times.

### Post-processing on partially contaminated bins

We defined genome bins with completeness higher than 50% and contamination higher than 10% as partially contaminated bins. A post-processing step was designed to clean these partially contaminated bins by re-clustering contigs within each contaminated bin using the Leiden algorithm. As the number of groups within each bin was expected to be small, the resolution parameter was kept to be 1 in the post-processing step. By this means, groups of relatively smaller bins, denoted by sub-bins, could be obtained and those sub-bins satisfying the minimum bin size requirement were retained and subsequently evaluated for their quality by CheckM.

### Comparison to other metagenomic binning pipelines

We compared HiCBin to all the other Hi-C-based metagenome deconvolution pipelines, i.e, ProxiMeta [20], MetaTOR (v0.1.7) [21], and bin3C (v0.1.1) [22]. We also compared HiCBin with MataBAT2 (v2.12.1) [33], which was a conventional binning pipeline using shotgun libraries only and achieved one of the best binning performance in the CAMI Challenge Datasets [53]. MetaTOR, bin3C, and MetaBAT2 are three open-source tools. MetaTOR was implemented with default parameters, followed by the recursive Louvain clustering [21]. bin3C and MetaBAT2 were run with default parameters. As ProxiMeta is a proprietary metagenomic genome binning platform without an open-source pipeline, we reanalyzed two datasets processed by ProxiMeta kit (i.e., the human gut and the wastewater datasets), and compared the CheckM validations of HiCBin to the results of ProxiMeta provided in their supplementary data. We also compared the quality of retrieved genome bins of HiCBin and the other three open-source binning softwares on the human gut sample. For the wastewater sample, as the restriction enzymes employed in the proprietary preparation kit were unspecified in the experiment, bin3C could not compute the number of restriction sites on contigs and normalize the raw contacts, resulting in the inapplicability of bin3C. Therefore, we additionally compared the HiCBin to the MetaTOR and MetaBAT2.

## Supplementary Information

**Additional file 1** Evaluation criteria of the clustering results, supplementary figures and tables. (PDF 140 KB). Three evaluation criteria of the clustering results, additional figures S1-S2 and table S1 supporting the manuscript.

**Additional file 2** The human gut CheckM report for HiCBin. (CSV 25 KB). The CheckM validation of draft genomic bins recovered from the human gut sample by HiCBin.

**Additional file 3** The human gut CheckM report for MetaBAT2. (CSV 20 KB). The CheckM results of draft genomic bins resolved from the human gut sample by MetaBAT2.

**Additional file 4** The human gut CheckM report for MetaTOR. (CSV 8 KB). The CheckM evaluation of draft genomic bins retrieved from the human gut sample by MetaTOR.

**Additional file 5** The wastewater CheckM report for HiCBin. (CSV 93 KB). The CheckM validation of draft genomic bins recovered from the wastewater sample by HiCBin.

**Additional file 6** The wastewater CheckM report for MetaTOR. (CSV 8 KB). The CheckM evaluation of draft genomic bins resolved from the wastewater sample by MetaTOR.

**Additional file 7** The wastewater CheckM report for MetaBAT2. (CSV 33 KB). The CheckM results of draft genomic bins retrieved from the wastewater sample by MetaBAT2.

**Additional file 8** Review history

## Data Availability

The HiCBin software is freely available at https://github.com/dyxstat/HiCBin under the GNU General Public License version v3 [54] and the source code used in the manuscript has been deposited in archived format at 10.5281/zenodo.5791459 [55]. All the datasets used in this study are publicly available from the NCBI Sequence Read Archive database (http://www.ncbi.nlm.nih.gov/sra). The synthetic metagenomic yeast sample was downloaded under the accession numbers: shotgun library SRR1263009, Hi-C library SRR1262938 [56]. The human gut sample is available through the accession numbers: shotgun library SRR6131123, Hi-C libraries SRR6131122 and SRR6131124 [57]. The accession numbers of the wastewater sample are SRR8239393 for shotgun library and SRR8239392 for Hi-C library [58]. CheckM validation results of the human gut sample and the wastewater sample for ProxiMeta are available at 10.1101/198713 [20] and 10.1038/s41396-019-0446-4 [28], respectively. Supporting material of bin3C used in comparison is available at 10.1186/s13059-019-1643-1 [22].
