# Supplementary material for: HiCBin: binning metagenomic contigs and recovering metagenome-assembled genomes using Hi-C contact maps
Source: Genome Biol. 2022 Feb 28;23:63. doi: 10.1186/s13059-022-02626-w (PMC8883645; doi:10.1186/s13059-022-02626-w)
Supplement: Supplementary file 1 — Additional file 1 Evaluation criteria of the clustering results, supplementary figures and tables. (PDF 140 KB). Three evaluation criteria of the clustering results, additional figures S1-S2 and table S1 supporting the manuscript. [file 13059_2022_2626_MOESM1_ESM.pdf]

Evaluation criteria of the clustering results

The Fowlkes-Mallows score

The Fowlkes-Mallows score (F-score) is defined as the geometric mean of the precision and recall, i.e,

$$\mathrm{FM} = \sqrt{\frac{TP}{TP+FP} \cdot \frac{TP}{TP+FN}}, \tag{S1}$$

where TP is the number of true positives, FP is the number of false positives, and FN is the number of false negatives.

The Adjusted Rand Index

The rand index (RI) is defined as the percentage of correct decisions made by the clustering algorithm, i.e.,

$$\mathrm{RI} = \frac{TP+TN}{TP+TN+FP+FN}, \tag{S2}$$

where TP is the number of true positives, TN is the number of true negatives, FP is the number of false positives, and FN is the number of false negatives.

Then, the Adjusted Rand Index (ARI) can be defined as

$$\mathrm{ARI} = \frac{\mathrm{RI} - \mathbb{E}(\mathrm{RI})}{\max(\mathrm{RI}) - \mathbb{E}(\mathrm{RI})}, \tag{S3}$$

where $\mathbb{E}(\mathrm{RI})$ denotes the Expected Rand Index.

The Normalized Mutual Information

Let U and V denote the sets of true class labels and predicted cluster labels, respectively. Define the entropy of a label set S as

$$H(S) = -\sum_{i=1}^{|S|} P(i)\log(P(i)), \tag{S4}$$

where $P(i) = |S_i|/N$ is the probability of an object in class S_i.

The mutual information (MI) between U and V is calculated by:

$$\mathrm{MI}(U,V) = \sum_{i=1}^{|U|}\sum_{j=1}^{|V|} P(i,j)\log(\frac{P(i,j)}{P(i) \times P'(j)}), \tag{S5}$$

where $P(i,j) = |U_i \cap V_j|/N$, $P(i) = |U_i|/N$, and $P'(j) = |V_j|/N$.

Then, the Normalized Mutual Information (NMI) is defined as

$$\mathrm{NMI}(U,V) = \frac{2 \times \mathrm{MI}(U,V)}{H(U)+H(V)}. \tag{S6}$$

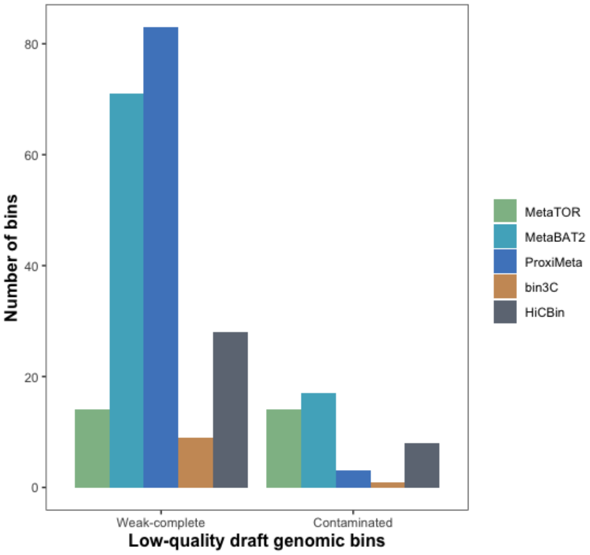

Fig S1: Comparison of low-quality draft genomic bins retrieved by different binning pipelines on the human gut dataset. MetaBAT2 created 71 weak-complete and 17 contaminated bins; MetaTOR generated 14 weak-complete and 14 contaminated bins; ProxiMeta recovered 83 weak-complete and 3 contaminated bins; bin3C constructed 9 weak-complete and 1 contaminated bins; HiCBin retrieved 28 weak-complete and 8 contaminated bins.

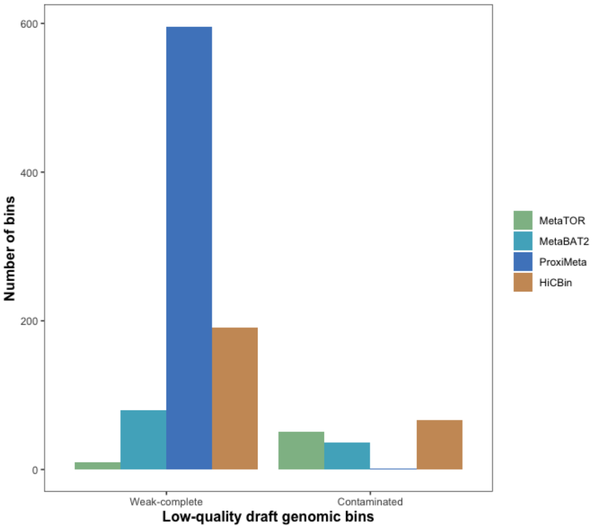

Fig S2: Comparison of low-quality draft genomic bins retrieved by different binning pipelines on the wastewater dataset. MetaBAT2 created 80 weak-complete and 36 contaminated bins; MetaTOR generated 10 weak-complete and 51 contaminated bins; ProxiMeta constructed 596 weak-complete and 1 contaminated bins; HiCBin retrieved 191 weak-complete and 67 contaminated bins.

Table S1: Reference genomes of the species list in the metagenomic yeast sample.

Genus	Species	Strain in sample	Reference strain
Saccharomyces	*cerevisiae*	FY4H	FY
Saccharomyces	*cerevisiae*	CEN.PK	CEN.PK
Saccharomyces	*cerevisiae*	RM11-1A	RM11-1A
Saccharomyces	*cerevisiae*	SK1	SK1
Saccharomyces	*paradoxus*	YDG613	
Saccharomyces	*mikatae*	FM356	IFO 1815
Saccharomyces	*kudriavzevii*	FM527	IFO 1802
Saccharomyces	*bayanus var. uvarum*	YZB5-113	CBS 7001
Naumovozyma	*castellii*	4310	NRRL Y-12630
Lachancea	*waltii*	Kwaltii ura3	NRRL Y-8285
Lachancea	*kluyveri*	FM628	CBS 3082
Kluyveromyces	*lactis*	MW98-8C	NRRL Y-1140
Kluyveromyces	*wickerhamii*	Y-8286	UCD 54-210
Ashbya	*gossypii*	WT	ATCC 10895
Scheffersomyces	*stipitis*	Y-11545	CBS 6054
Pichia	*pastoris*	JC308	GS115
