# Supplementary material for: HiCBin: binning metagenomic contigs and recovering metagenome-assembled genomes using Hi-C contact maps
Source: Genome Biol. 2022 Feb 28;23:63. doi: 10.1186/s13059-022-02626-w (PMC8883645; doi:10.1186/s13059-022-02626-w)
Supplement: Supplementary file 8 — Additional file 8 Review history [file 13059_2022_2626_MOESM8_ESM.docx]

**1^st^ round**

**Reviewer 1**

In this paper, the authors presented HiCBin, a computational method to bin metagenomic assemblies using Hi-C contact maps. There are a couple of similar methods in the field. In this study, the authors addressed two key issues in this field: normalization and clustering. The improvements made in this method allow HiCBin to perform better than existing methods, demonstrated by a Mock metagenomic datasets and two real metagenomic datasets. The manuscript was well written with enough details.

Below are some comments and questions.

About HiCzin: the first time HiCzin was mentioned, I thought it might be a typo. But it is another independent method by the authors published earlier. So it is better to introduce HiCzin and include a citation when HiCzin was first mentioned.

In the analysis, TAXAassign was used to assign taxa to contigs by aligning the contigs to reference sequences. So basically, the contigs were already binned by TAXAassign. Due to the use of TAXAassign, HiCBin is not really an independent binning method. A few questions here: (1) TAXAassign aligns contig to the NCBI nt database, so it can be quite time-consuming. (2) Did other binning methods being compared also used TAXAassign or similar methods to align the contigs to reference sequences? (3) please discuss whether the improved performance is due to better algorithms for analyzing Hi-C sequence or due to the fact these contigs were already binned by TAXAassign.

Figure 2a and 3a. I feel there can be better plots to show the data. Also, data for other methods are not shown. So figures are not informative.

In figures 2b and 3b, the bins with > 10% contamination or < 50% completeness should also be included. So that readers can also compare low-quality bins.

Table 5, does the compute time for HiCBin include TAXAassign? If not, the time used in TAXAassign should also be listed.

**Reviewer 2**

The paper by Du and Sun deals with the problem of binning metagenomics contigs using Hi-C spatial proximity data. Metagenomics contigs can be easily obtained via short-read assembly: the problem addressed here is to use Hi-C data to bin or cluster those contigs into groups that are likely to belong to the same species (i.e., binning).

The method proposed by Du and Sun, called HiCBin, uses the Leiden algorithm for the clustering step (based on the Potts spin-glass model). This algorithm was selected because it provided superior performance on the yeast dataset (on which the ground truth assignment is known). Different methods for the normalization of the Hi-C data have been explored on the same dataset (HiCzin appears marginally better than others).

Experimental results on real datasets are reported on two additional datasets, namely human guts and wastewater metagenomic samples; due to the lack of a ground truth on these datasets,

the authors checked for the presence of marker genes in the contigs to determine whether contigs were considered "near-complete", "substantially complete" or "moderately complete". According to this measure their method HiCBin performs significantly better than MetaTOR, MetaBAT2, Bin3C, and ProxiMeta.

strengths

* This is an important problem; tools in this domain are expected to have a significant impact on the analysis of metagenomics data

* It is well-written paper (with some exceptions, see minor issues below)

* The choice of specific methods in the analysis pipeline is well justified

* There are sufficient details to allow replication of the experiments

weaknesses

* The major weakness of this work is the measure used to determine the quality of the experimental results for the human and wastewater samples: the proposed

CheckM criteria for completeness seems very crude and incomplete because many contigs won't have marker genes. so they cannot be properly evaluated;

I do not know what other type of quality metric to use to evaluate the results, but I am not totally convinced by the experimental results based on CheckM

* The algorithmic novelty is quite limited - the proposed method uses a set of already developed ideas in a novel pipeline

* The Github repository is well organized but it does not contain any link to short read data (metagenomics and Hi-C) to test the pipeline

minor issues

* page 2, "generating tremendous read libraries". I am not sure "tremendous" is the appropriate term here

* scientific species names should be in italics, e.g., page 10, S. cerevisiae and the names in Figure 1, and supplemental figure S1

* some sentences are awkward or vague, e.g., "In the future, it is better to explore the binning performance with finer resolution. " (page 15). "Therefore, it is worthy of further research on the evaluation of MAG retrieval." (page 15) or "High-throughput chromosome conformation capture (Hi-C) [16] is a DNA proximity technique displaying a great potential to break through the barriers in the metagenomic binning domain." (page 2 -- "displaying a great potential"? what "barriers"?)

**Authors’ response**

**Reviewer 1**

We sincerely appreciate your helpful and constructive comments! Your suggestions have significantly helped us improve our manuscript. In the revision, we have followed your comments and addressed all the concerns in detail as follows.

**1. The first time HiCzin was mentioned, I thought it might be a typo. But it is another independent method by the authors published earlier. So it is better to introduce HiCzin and include a citation when HiCzin was first mentioned.**

**Response:** Thanks, we added the citation when HiCzin was first mentioned in the introduction section (page 3).

**2. TAXAassign aligns contig to the NCBI nt database, so it can be quite time-consuming.**

**Response:** We agree with the reviewer that assigning contigs to the NCBI nt database is time consuming. Therefore, we recorded the running time of TAXAassign and presented the time consumed by TAXAassign in the section ‘*Running time of HiCBin, MetaBAT2, and bin3C*’ (page 17). Specifically, the TAXAassign software was run on a machine with 4-way 6-core 1.87 GHz Intel Xeon CPUs and spent 6.5h, 7.5h, and 17.7h annotating contigs assembled from the synthetic yeast, human gut, and wastewater datasets, respectively. Though HiCBin requires an extra preprocessing step on running the TAXAassign software and thus takes extra time compared to the standard Hi-C-based analysis pipeline, the time consumed by TAXAassign is acceptable, especially when compared to the time needed for the assembly of long contigs from short reads, which always requires much more time on real metagenomic datasets. For example, MEGAHIT spent about 104h assembling contigs while it took 17.7h for TAXAassign to annotate contigs for the same dataset (page 18).

**3. Did other binning methods being compared also used TAXAassign or similar methods to align the contigs to reference sequences?**

**Response**: No.

**4. Please discuss whether the improved performance is due to better algorithms for analyzing Hi-C sequence or due to the fact these contigs were already binned by TAXAassign.**

**Response:** Thanks for your important comment! Firstly, HiCBin does not directly use the contig labels annotated by TAXAassign for clustering contigs. There are two functions for the contig labels: generating the intra-species Hi-C interactions in the normalization step and determining the resolution parameter for the Leiden algorithm. Therefore, we think that the contig labels don’t have a direct impact on the final clustering results and the improvement of binning performance for HiCBin is due to the better algorithms we use instead of the known assignment of these contigs.

Moreover, we have conducted a new experiment to consolidate our statement. Specifically, we evaluated the clustering performance of contigs that were not annotated by TAXAassign on the metagenomic yeast dataset. As shown in Table 1, HiCBin still greatly outperformed both shotgun-based and Hi-C-based binning tools for clustering contigs without any prior information, which indicated that the improved binning performance was due to better algorithms of HiCBin for analyzing metagenomic Hi-C data. Results of the new experiment were also presented in the revised manuscript (page 11).

|  | F-score | ARI | NMI |
| --- | --- | --- | --- |
| MetaBAT2 | 0.639 | 0.512 | 0.675 |
| bin3C | 0.670 | 0.581 | 0.691 |
| HiCBin | **0.935** | **0.924** | **0.890** |

Table 1. Comparison of the performance of Hi-C-based binning pipelines: HiCBin and bin3C, and a shotgun-based binning tool MetaBAT2 based on contigs that were not annotated by TAXAassign.

**5. Figure 2a and 3a. I feel there can be better plots to show the data. Also, data for other methods are not shown. So figures are not informative.**

**Response:** Thanks for your comment. Figure 2a and 3a in the original manuscript just follow the plotting style of Figure 6 in the bin3C paper (DeMaere et al. Genome Biology 2019) and Figure 2c in the ProxiMeta paper (Press et al. bioRxiv 2017) to present the quality of draft genomic bins according to the CheckM criteria. To make the plots clearer, we have added some explanations in the captions as ‘*Completeness (black) and contamination (red) of draft genomic bins retrieved by HiCBin on the human gut dataset. The x-axis shows the ranks of bins according to their completeness’* (page 14: Figure 2; page 16: Figure 3)*.*

Moreover, we have followed your helpful suggestion and plotted the similar figures for other binning tools for comparison. We presented all figures in the main text of the revised manuscript (page 14: Figure 2b-f; page 16: Figure 3b-e).

**6. In figures 2b and 3b, the bins with > 10% contamination or < 50% completeness should also be included. So that readers can also compare low-quality bins.**

**Response:** Thanks for this helpful comment. We have followed your suggestion. Besides the original high-quality draft genomic bins, we define two categories of low-quality draft genomes, i.e., weak-complete (≥ 20% and <50% completeness, ≤ 10% contamination) and contaminated (≥ 20% completeness, ≥ 10% contamination) on page 8. Results of low-quality draft genomic bins are presented in the Supplementary Material Figures S1 and S2. We also add some analyses on the results of low-quality bins for both real samples (page 13 and page 15).

**7. Table 5, does the compute time for HiCBin include TAXAassign? If not, the time used in TAXAassign should also be listed.**

**Response:** No. As TAXAassign is a preprocessing step for HiCBin, the computing time for HiCBin does not include TAXAassign. We have presented the time consumed by TAXAassign in the section ‘*Running time of HiCBin, MetaBAT2, and bin3C*’ (page 17) in the revised manuscript.

**Reviewer 2**

We sincerely appreciate your helpful and constructive comments! Your suggestions have significantly helped us improve our manuscript. In the revision, we have followed your comments and addressed all the concerns in detail as follows.

**1. The major weakness of this work is the measure used to determine the quality of the experimental results for the human and wastewater samples: the proposed CheckM criteria for completeness seems very crude and incomplete because many contigs won't have marker genes. so they cannot be properly evaluated; I do not know what other type of quality metric to use to evaluate the results, but I am not totally convinced by the experimental results based on CheckM.**

**Response:** Thanks for your important comment. We agree on your point and admit that whether the marker-gene based validation could accurately reflect the true completeness and contamination of recovered MAGs deserves further exploration. We also add your thoughtful comment to the discussion section in the revised manuscript (page 18). However, to the best of our knowledge, the CheckM criterion is still the most popular method to determine the quality of bins on real samples and has multiple merits. In our manuscript, HiCBin is compared to all three Hi-C-based binning methods (i.e, MetaTOR (Baudry et al. Front. Genet. 2019), ProxiMeta (Press et al. bioRxiv 2017), and bin3C (DeMaere et al. Genome Biology 2019)) and one popular shotgun-based binning tool (i.e, MetaBAT2 (Kang et al. PeerJ 2019)). These papers also validated their results using the CheckM criteria for completeness on real datasets.

Moreover, to consolidate our validations on real samples, we provided additional metrics to evaluate the clustering quality using the contigs annotated by TAXAassign in the normalization step. Specifically, we randomly divided the labeled contigs into two equal parts. One part was utilized to construct the intra-species contacts and determine the resolution parameter by HiCBin while the other part served as the ground truth to evaluate the binning performance of different software packages in term of F-score, ARI, and NMI (page 8). We then applied the new metric to two real samples.

For the human gut dataset, 3,113 contigs out of a total of 6,225 labeled contigs were randomly selected out as the ground truth to do the evaluation while the remaining 3,112 contigs served as the input of HiCBin. Since MetaTOR splits the long contigs into shorter pieces referred to as chunks and then clusters these chunks while other binning methods bin the assembled contigs, it is not valid to compare MetaTOR to other binning pipelines without splitting in terms of F-score, ARI, and NMI. Moreover, ProxiMeta is proprietary without the open-source software. Therefore, we compared HiCBin to the remaining two binning tools, i.e., one shotgun-based binning method MetaBAT2 and one Hi-C-based binning pipeline bin3C (Table 2). HiCBin still outperformed both binning methods, which was consistent with the CheckM validation.

|  | F-score | ARI | NMI |
| --- | --- | --- | --- |
| MetaBAT2 | 0.751 | 0.729 | 0.782 |
| bin3C | 0.709 | 0.691 | 0.775 |
| HiCBin | **0.816** | **0.800** | **0.824** |

Table 2. Comparison of the binning performance of MetaBAT2, bin3C, and HiCBin using half of the contigs that were labeled by TAXAassign in terms of F-score, ARI, and NMI on the human gut sample.

For the wastewater dataset, we randomly selected out 17,481 labeled contigs from a total of 34,961 contigs annotated by TAXAassign and these contigs served as the ground truth for the binning evaluation. The remaining half of labeled contigs were utilized to generate the intra-species Hi-C interactions in the normalization step and determine the resolution parameter in the clustering step by HiCBin. The binning performance of MetaTOR and ProxiMeta could not be evaluated by this method due to the same reasons explained in the section of analyzing the human gut sample. Moreover, bin3C required the input of the names of restriction enzymes as it normalized the raw Hi-C contacts using the number of restriction sites on contigs. Since the enzymes utilized in the experiment were unknown, bin3C was not applicable to this dataset. Therefore, we evaluated the bins retrieved by HiCBin and MetaBAT2 (Table 3) and HiCBin outperforms MetaBAT2 in terms of F-score, ARI and NMI.

|  | F-score | ARI | NMI |
| --- | --- | --- | --- |
| MetaBAT2 | 0.585 | 0.568 | 0.824 |
| HiCBin | **0.721** | **0.708** | **0.870** |

Table 3. Comparison of the binning performance of MetaBAT2 and HiCBin using half of the contigs that were labeled by TAXAassign in terms of F-score, ARI, and NMI on the wastewater sample.

**2. The algorithmic novelty is quite limited - the proposed method uses a set of already developed ideas in a novel pipeline.**

**Response:** As the reviewer mentioned, Hi-C-based binning is a very important problem since the accurate retrieval of MAGs is vital to downstream analyses, such as probing the virus-host interactions. However, to the best of our knowledge, this problem has not been well resolved so far. Firstly, normalization methods in publicly available Hi-C-based binning pipelines cannot correct all explicit biases. We proved that experimental biases of Hi-C contacts were harmful to the binning performance in our HiCBin manuscript. Secondly, though multiple clustering algorithms have been applied to the contig clustering based on the Hi-C contact maps, DeMaere et al. have previously found that those clustering algorithms were not sufficiently effective (DeMaere et al. PeerJ 2016). Finally, spurious inter-species contacts weakened the interpretability of the Hi-C data. However, none of available Hi-C-based binning tools attempted to detect and remove the spurious contacts. Therefore, there is an urgent need for solving these problems and we put forward a novel Hi-C-based binning pipeline based on the most advanced normalization and clustering methods. We have proved that HiCBin could solve all the above problems. HiCBin is also the first pipeline to introduce the spurious contact detection into binning and we have proved that removing spurious contacts could indeed improve the performance of binning. We hope the improved binning performance using our new pipeline could assist in the downstream metagenomic analysis for microbiologists.

**3. The Github repository is well organized but it does not contain any link to short read data (metagenomics and Hi-C) to test the pipeline.**

**Response:** Thanks for your comment. We have added the link to the testing short read data on the GitHub repository (‘*0. Download the shotgun and Hi-C libraries’* in the section ‘*Initial data preparation*’). Moreover, we have provided a folder containing all testing input data for HiCBin software package on the GitHub repository (https://github.com/dyxstat/HiCBin/tree/main/test). Users can use these data to test both the data preparation pipeline and the HiCBin software package conveniently.

**4. page 2, "generating tremendous read libraries". I am not sure "tremendous" is the appropriate term here.**

**Response:** Thanks, we revised this phase as follows:

*generating huge amounts of short reads.*

We also re-read the manuscript again and did our best to correct other spelling or grammar errors.

**5. scientific species names should be in italics, e.g., page 10, S. cerevisiae and the names in Figure 1, and supplemental figure S1.**

**Response:** Fixed, thanks.

**6. some sentences are awkward or vague:**

**"In the future, it is better to explore the binning performance with finer resolution. " (page 15).**

**Response:** Thanks for the comment. We have rewritten this sentence to make the meaning clearer (page 18):

*In the future, it is necessary to explore the contig binning at the strain level.*

**"Therefore, it is worthy of further research on the evaluation of MAG retrieval." (page 15)**

**Response:** Thanks for the comment. We have rewritten this sentence as follows (page 18):

*Though CheckM is the most popular method to evaluate the quality of bins on real samples, how accurately the marker-gene based validation could reflect the true completeness and contamination of recovered MAGs deserves further exploration considering that some genomic regions do not contain marker genes.*

**"High-throughput chromosome conformation capture (Hi-C) [16] is a DNA proximity technique displaying a great potential to break through the barriers in the metagenomic binning domain." (page 2 -- "displaying a great potential"? what "barriers"?)**

**Response:** Thanks for the helpful comment. We have modified the vague expression and explained what the ‘barrier’ is in the revised sentence as follows (page 2):

*High-throughput chromosome conformation capture (Hi-C) [16] is a DNA proximity ligation technique having the capacity to retrieve MAGs accurately from a single sample.*

**2^nd^ round**

**Reviewer 1**

All my comments were adequately addressed and the revised manuscript is largely improved.

**Reviewer 2**

The authors have satisfactorily answered to all my questions. In particular, the additional experiments in which they randomly divide the labeled contigs into two equal parts is convincing. I assume that the random partition was carried out multiple times and the reported performance is an average. If so, it would have been good to know the variance of the performance among difference partitions.

**Authors’ response**

**1. The authors have satisfactorily answered to all my questions. In particular, the**

**additional experiments in which they randomly divide the labeled contigs into two equal**

**parts is convincing. I assume that the random partition was carried out multiple times**

**and the reported performance is an average. If so, it would have been good to know**

**the variance of the performance among difference partitions.**

**Response:** Thanks for your helpful comment! We have conducted the validation

experiments ten times and added the standard deviations of the performance among

difference partitions to Tables 5 and 6 in the manuscript (Page 7 and 11).
